# Improving clinical outcomes and patient satisfaction among patients with coronary artery disease: an example of enhancing regional integration between a cardiac centre and a referring hospital

**DOI:** 10.1186/s12913-020-05352-w

**Published:** 2020-06-03

**Authors:** Dennis van Veghel, Mohamed Soliman-Hamad, Daniela N. Schulz, Bernard Cost, Timothy A. Simmers, Lukas R. C. Dekker

**Affiliations:** 1 Catharina Ziekenhuis, Michelangelolaan 2, 5623 EJ Eindhoven, The Netherlands; 2 SJG Weert, Vogelsbleek 5, 6001 BE Weert, The Netherlands

**Keywords:** Regional integration, Cardiac care, Coronary artery disease, Patient satisfaction, Value-based health care, Outcome measures, Clinical outcomes

## Abstract

**Background:**

Value-based healthcare (VBHC) is a promising strategy to increase patient value. For a successful implementation of VBHC, intensive collaborations between organizations and integrated care delivery systems are key conditions. Our aim was to evaluate the effects of a pilot study regarding enhancing regional integration between a cardiac centre and a referring hospital on patient-relevant clinical outcomes and patient satisfaction.

**Methods:**

The study population consisted of a sample of patients treated for coronary artery disease by use of a coronary artery bypass graft (CABG) or a percutaneous coronary intervention between 2011 and 2016. Since 2013, the two hospitals have implemented different interventions to improve clinical outcomes and the degree of patient satisfaction, e.g. improvement of communication, increased consultant capacity, introduction of outpatient clinic for complex patients, and improved guideline adherence. To identify intervention effects, logistic regression analyses were conducted. Patients’ initial conditions, like demographics and health status, were included in the model as predictors. Clinical data extracted from the electronic health records and the hospitals’ cardiac databases as well as survey-based data were used.

**Results:**

Our findings indicate a non-significant increase of event-free survival of patients treated for coronary artery disease between 2014 and 2016 compared to patients treated between 2011 and 2013 (97.4% vs. 96.7% respectively). This non-significant improvement over time has led to significant better outcomes for patients referred from the study referring hospital compared to patients referred from other hospitals. The level of patient satisfaction (response rate 32.2%; 216 out of 669) was improved and reached statistically significant higher scores regarding patient information and education (*p* = .013), quality of care (*p* = .007), hospital admission and stay (*p* = .032), personal contact with the physician (*p* = .024), and total impression (*p* = .007).

**Conclusions:**

This study shows a promising effect of regional integration. An intensified collaboration in the care chain, organized in a structured manner between a cardiac centre and a referring hospital and aiming at high quality, resulted in successful improvement of clinical outcomes and degree of patient satisfaction. The applied method may be used as a starting point of regional integration with other referring hospitals. We encourage others to organize the whole care chain to continuously improve patient-relevant outcomes and patient satisfaction.

**Trial registration:**

ISRCTN11311830. Registered 01 October 2018 (retrospectively registered).

## Background

Worldwide, value-based healthcare (VBHC) initiatives are implemented by healthcare providers to achieve high value for patients [1, 2]. Patient value is defined as the achieved health outcomes divided by healthcare delivery costs [3]. According to Porter, a limited set of outcome measures that matter most to patients should be selected. The outcome measures hierarchy [4] is a tool to help identifying outcome measures that matter most to patients and at the same time to cover all relevant tiers: outcomes regarding health status, process of recovery and sustainability of health. These outcomes measures make it possible to measure quality of performance. In the Netherlands, the *‘Meetbaar Beter’* foundation [5] started to play a role in implementing VBHC in cardiac care in 2011, using standard sets of outcome measures that are aligned with the sets developed by the International Consortium for Health Outcomes Measurement (ICHOM) [6].

Another performance measure is patient satisfaction. Patient satisfaction is often related to outcomes and can be described as an indirect or proxy indicator of the quality of doctor or hospital performance [7]. Donabedian [8, 9] has noted that patient satisfaction is not only an important component of quality of care, but also a strong contributor to the definition of quality from the perspective of clients’ values and expectations. Different studies have shown that satisfied patients are more likely to better comply with providers’ medical regimens and orders, to continue using medical care services and to cooperate or maintain relationship with specific providers when compared to unsatisfied patients [8, 10–12]. Besides, associations have been found between patient satisfaction and outcomes, such as readmissions [13, 14].

Since outcomes and patient satisfaction are influenced by various specialties and interventions in the treatment process of a patient, it is recommended to integrate care delivery systems at a regional level [4]. Delivering care across separate facilities and expanding excellent services across geographies are key components of the strategic agenda for the implementation of VBHC [1]. This means that regional integration, defined as working together across disciplines and institutions (for example by sharing knowledge, outsourcing activities, and organizing the full cycle of care) is a key condition for a successful implementation of VBHC.

In the Netherlands, there are 79 hospitals of which 16 accommodate cardiac centres. These cardiac centres hold licenses allowing them to perform complex cardiac procedures by law (*Wet op de Bijzondere Medische Verrichtingen, WBMV*) [15]. The Catharina Hospital in Eindhoven is one of the 16 cardiac centres, performing approximately 7000 complex cardiac interventions per year. Almost 70% of these patients are referred by hospitals in the region around Eindhoven. The bigger part of the pre- and post-operative care takes place at the referring hospital, as patients are transferred back to the referring hospital within a few days after the intervention. Due to this referral process, optimizing the care chain is a prerequisite for excellent outcomes and high patient satisfaction. The “St. Jans Gasthuis” (SJG) in Weert is one of the referring hospitals of the Catharina cardiac centre. Yearly, about 9% of all patients are referred from this hospital for undergoing a heart intervention in the Catharina cardiac centre. Since 2013, the Catharina cardiac centre and the SJG Weert intensified their collaboration. Interventions had been implemented to improve clinical outcomes and patient satisfaction.

The aim of the present report is to analyze the effects of a pilot study regarding enhancing regional integration between two hospitals on patient-relevant outcomes and patient satisfaction.

## Methods

### Study design, patients and inclusion criteria

An observational cohort study design was adopted. We used the following inclusion criteria: patients diagnosed with coronary artery disease (CAD), referred from another hospital in the region and treated by use of a coronary artery bypass graft (CABG) or a percutaneous coronary intervention (PCI) in the Catharina cardiac centre between 01/01/2011–31/12/2016.

For the first part of the outcome analysis, we used the following two cohorts: The baseline cohort included patients referred from SJG Weert and treated in the Catharina cardiac centre during the period from 2011 through 2013 (*n* = 820). The evaluation cohort included patients referred from SJG Weert and treated in the Catharina cardiac centre between 2014 and 2016 (*n* = 655).

For the second part of the outcome analysis, all patients referred to the Catharina cardiac centre for a CABG or PCI between 2011 and 2016 were included (*n* = 12,013). We subdivided the cohort into patients referred from SJG Weert (also split into baseline cohort 2011–2013 and evaluation cohort 2014–2016) and patients referred from all other hospitals to the Catharina cardiac centre (also split into baseline cohort 2011–2013 and evaluation cohort 2014–2016).

### Clinical outcomes

To analyze outcomes for patients with CAD who underwent CABG or PCI, the following outcome measures, with a clinically relevant follow-up duration up to a maximum of 120 days, are used: 30-day mortality, 120-day mortality, cerebrovascular accident (CVA) within 72 h, deep sternal wound infection (DSWI) within 30 days, surgical re-exploration within 30 days, urgent CABG within 24 h, and myocardial infarction (MI) within 30 days. For patients treated with a PCI, 30-day mortality is used whereas for patients treated with CABG, 120-day mortality is used. This choice is based on previous research that has shown that all cardiac surgery-related mortalities were covered at 120 days post-surgery [16] whereas for PCI, risk of death seems to move from cardiac to non-cardiac after a period of 30 days post-PCI [17]. For all outcome measures, definitions and time periods as defined by *Meetbaar Beter* [18] are adopted in the present study.

In addition to the separate outcome measures, all outcomes have been combined into the variable “event-free survival” (i.e. no mortality within 120-days (CABG) and 30-days (PCI) respectively), no complications (i.e., none of the before mentioned outcomes) and no MI within 30 days after the intervention).

Outcomes have been retrieved from the electronic health record and cardiac databases used in the Catharina cardiac centre and in SJG Weert.

### Patient satisfaction

To measure patient satisfaction, self-administered questionnaires were used (see additional file 1). The sample consisted of patients referred from SJG Weert to Catharina cardiac centre in the year 2013 and in the period January until September 2015. They received questionnaires delivered by postal mail. In the cover letter, we explained that participation was voluntarily and that privacy was guaranteed. The questionnaires could be answered on paper and sent back by post or filled in online via internet. The questionnaires were completed anonymously and thus no link could be made between answers on the questions and personal information of the patients. A total of 28 items were included to assess the following topics: communication with the hospital (2 items); communication between the hospitals and the patient’s general practitioner (2 items); education and education material (4 items); consistency/compatibility between the two hospitals (2 items); access time (2 items); quality of care (4 items); unexpected events and complications (3 items); hospital stay (4 items); and personal contact with physician in both hospitals (2 items). On a scale from 1 to 10, patients were asked “To what extent are you satisfied with …” , followed by the specific item. All questions were assessed separately regarding both hospitals. Patients were asked to give an overall grade of the delivered care in both hospitals on a scale from very bad (=1) to excellent (=10).

### Interventions to improve the care chain

Catharina cardiac centre and SJG Weert organized a shared project to identify improvement possibilities in the care chain. In this shared project, led by a steering group with representatives of both hospitals, interventions were selected and implemented at both hospitals to improve quality of care in both hospitals and in the referring process between the hospitals. The following interventions have been implemented since 2013:

- Information and communication: improvement of the communication within and between both hospitals regarding patients referred to and discussed in the heart team meetings; a new protocol for patients’ discharge in Catharina cardiac centre and SJG Weert, modifying patient brochures in both hospitals to better adhere to each other.
- Knowledge transfer on daily basis by introducing a daily discussion session regarding hospitalized patients for the entire consultant team. Also frequent multidisciplinary meetings to discuss complex patients were introduced.
- Consultant resources: the consultant capacity in SJG Weert was increased from 4 full-time equivalents (FTE) to 4,5 FTE. Time investment was made into in-patient care by separating supervision tasks for the emergency department and coronary care unit respectively the cardiology nursing ward. Also at the outpatient clinic, there was a modification of planning, leading to more time reserved for new patients. Supervision of the imaging department was improved by reserving time of an imaging-consultant, and on a routine basis an educational plan for employees of the imaging department was started.
- Care for complex patients: introduction of outpatient clinics prior to complicated procedures and for specific patient groups run by consultants from Catharina cardiac centre and a special attention to discussing high-risk patients.
- Improving guideline adherence: introduction of “time-outs” in the catheterization lab and change of discharge policy.

### Statistical analyses

Data were analyzed using SPSS software, version 23 (IBM Corp, Armonk, NY, USA). Descriptive statistics were used to describe the baseline characteristics and (uncorrected) outcomes for patients referred from SJG Weert between 2011 and 2013 (baseline cohort) and patients referred from SJG Weert between 2014 and 2016 (evaluation cohort). To be able to study the effects of this project and exclude effects of generic quality improvement projects in the Catharina Hospital, outcomes of patients of SJG Weert were also compared with outcomes of patients of other referring hospitals treated for CAD in the Catharina cardiac centre during the same period regarding “event-free survival”. Differences in outcomes between patients from SJG Weert compared to patients from all other referring hospitals at pre- and post-measurement were explored by means of logistic regression analyses using the top-down procedure. The dependent variable was event-free survival. Risk-adjustment was performed for the following patient characteristics: age, gender, diabetes, renal insufficiency, multi-vessel disease, LVEF and urgency of the procedure. To examine whether significant differences exist between patient satisfaction at baseline and 2 years later, the mean scores of the two groups on the different aspects were explored by means of independent samples t-tests. Tests were performed at alpha = .05.

## Results

### Clinical outcomes

In total, 1475 patients referred from SJG Weert to the Catharina cardiac centre for a treatment for CAD were included in the analyses. The baseline characteristics are shown in Table 1.

**Table 1:** Patient characteristics (coronary artery disease: PCI and CABG)

Variable	Baseline cohort 2011–2013	Evaluation cohort 2014–2016	p
Male gender	628 (76.6%)	509 (77.7%)	.610
Age, year, mean	65.6 ± 10.7	66.3 ± 10.8	.243
Diabetes	126 (15.4%)	97 (15.3%)	.947
Renal insufficiency	151 (18.5%)	117 (19.1%)	.772
Multivessel disease	455 (55.6%)	363 (55.7%)	.984
LVEF (< 50%)	88 (12.0%)	92 (16.4%)	.042
Non-elective procedure	405 (49.4%)	329 (50.2%)	.749

Data are presented as mean ± SD or number (%); *CABG* Coronary artery bypass grafting; *LVEF* Left ventricular ejection fraction; *PCI* Percutaneous coronary intervention

Table 2 presents the uncorrected clinical outcomes for the two cohorts. When combining both the PCI and the CABG groups, we observed an improvement in all outcomes (Table 2).

**Table 2:** Clinical outcome comparisons between the baseline cohort (2011–2013) and the evaluation cohort (2014–2016)

	Baseline cohort 2011–2013		Evaluation cohort 2014–2016	
	n	%	n	%
**CABG**	184		128	
120-day mortality	1	0.5	0	0.0
CVA	1	0,5	0	0.0
DSWI	3	1.6	1	0.8
Surgical re-exploration	10	5.4	6	4.7
**PCI**	636		527	
30-day mortality	9	1.4	5	0.9
Urgent CABG	2	0.3	0	0.0
MI	3	0.5	5	1.0
**Coronary artery disease**^a^	810		643	
Mortality	10	1.2	5	0.8
Complications	17	2.1	11	1.8
Event-free survival (short-term)	780	96.7	603	97.4

^a^ Treated with either CABG or PCI

As demonstrated in Table 3, the results of the logistic regression analysis show that event-free survival was statistically significantly higher in SJG Weert compared to that of patients of all other referring hospitals in 2014–2016. The difference in event-free survival between the hospitals was not statistically significant in 2011–2013.

**Table 3:** Results of the logistic regression analysis with event-free survival (0 = no event; 1 = event) as dependent variable among patients with coronary artery disease ^**1**^

	SJG Weert	Patients from all other referring hospitals	OR	p
2011–2013	96.7%	95.4%	1.05	.653
2014–2016	97.4%	95.1%	1.39	.046

^1^ Exclusion of patients who underwent a second procedure (PCI or CABG) within 120 days after the initial procedure

### Patient satisfaction

**Fig. 1:**
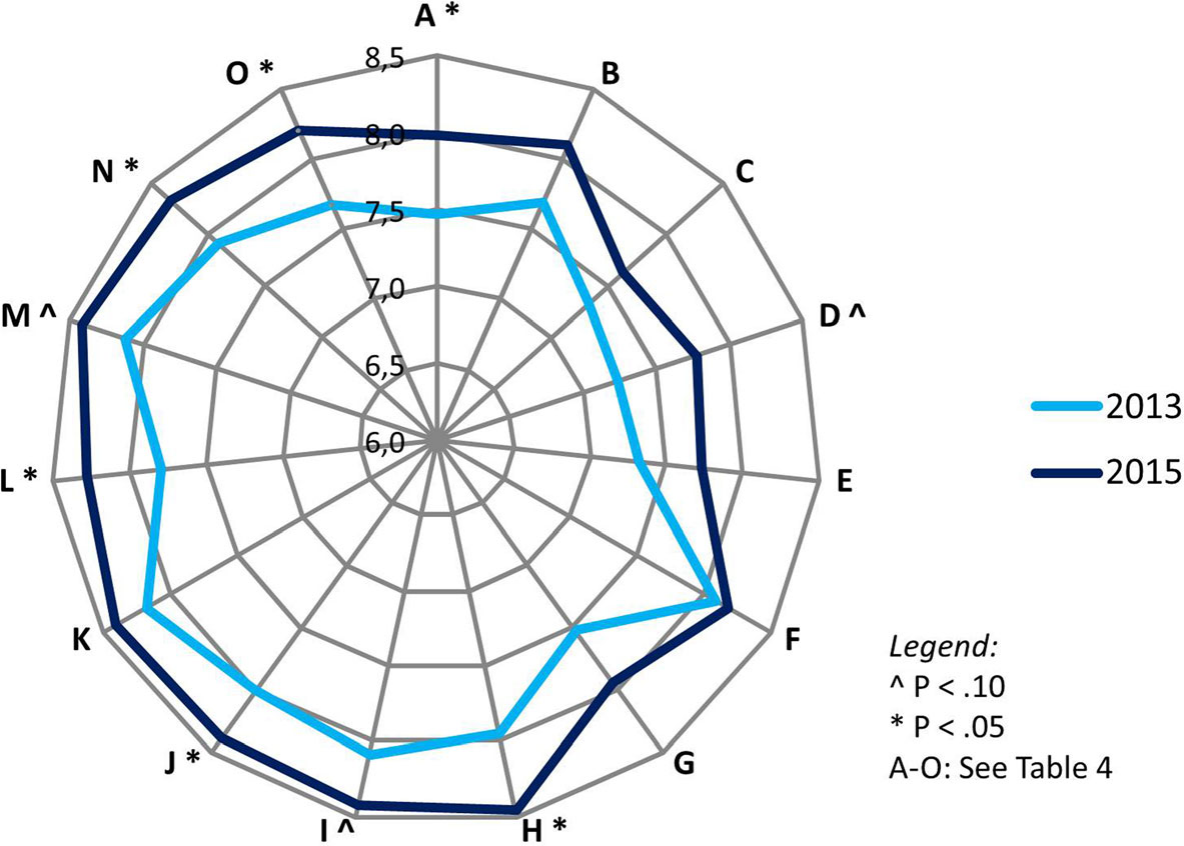
Results of patient satisfaction questionnaires

In total, 216 out of 669 patients (32.2%) completed the patient satisfaction questionnaire. The mean scores on both survey points are shown in Fig. 1. The score referring to education and education material, which was related to both hospitals, was significantly higher in 2015 compared to 2013. In the SJG Weert, the mean scores regarding the overall grade is significantly improved, and the scores on the specific items regarding quality of delivered care, hospital admission and the personal contact with the medical specialist were rated significantly higher in 2015 than in 2013. In the Catharina cardiac centre, the personal contact between patient and medical specialist seemed to be improved. As presented in Table 4, borderline significant differences (*p* < .10) were found regarding other aspects, too.

**Table 4:** Differences regarding patient satisfaction between patients treated in 2013 and patients treated in 2015

Patient satisfaction variable	2013 *n* = 108	2015 *n* = 108	p
A. Patient information and education	7.47	7.98	.013
B. Expectation management	7.69	8.09	.127
C. Alignment between both hospitals	7.33	7.62	.214
D. Communication with the GP (SJG Weert)	7.24	7.77	.086
E. Communication with the GP (Catharina)	7.33	7.73	.189
F. Duration to approach and pathway (SJG Weert)	8.09	8.18	.729
G. Duration to approach and pathway (Catharina)	7.53	7.95	.134
H. Quality of care (SJG Weert)	7.95	8.46	.007
I. Quality of care (Catharina)	8.08	8.43	.057
J. Admission and stay (SJG Weert)	8.00	8.39	.032
K. Admission and stay (Catharina)	8.17	8.41	.155
L. General mark (SJG Weert)	7.80	8.29	.007
M. General mark (Catharina)	8.13	8.42	.070
N. Personal contact between patient and physician (SJG Weert)	7.90	8.32	.024
O. Personal contact between patient and physician (Catharina)	7.67	8.20	.031

## Discussion

This report presents the effects in terms of clinical outcomes and patient satisfaction as a results of an intensified collaboration between the Catharina cardiac centre and the referring hospital SJG Weert. We described the measures taken to achieve this cooperation and as a result, improvement of both the clinical results and patient satisfaction have been noticed.

Regional integration of health care delivery systems is one of the key elements of VBHC. It is advised to organize patient pathways for patient groups with the same medical condition [4]. This requires new forms of collaboration between health care professionals and providers. In the Dutch health care system, mergers of hospitals have been observed over the last decade. However, these mergers are rarely successful in perspective of quality improvement [19]. In an earlier Dutch report [20], Roeg et al. concluded that intensive community-based care requires a highly complex organization, which is reflected by the diversity of the clusters. The emphasis on cooperation with other institutes is significant, and this should ideally be characterized as a chain of care [21]. This means that single services provided by separate institutes need to be strongly linked and that interorganisational and interdisciplinary service is essential for an intensive community-based care. The care chain includes care at both locations and the interaction between both hospitals. In our study, both hospitals jointly identified improvement potential and implemented the interventions at both locations, resulting in positive effects. Our study revealed better outcomes in terms of “event-free survival” for SJG Weert patients than for patients referred from other hospitals. The finding of this study encourages us to implement similar projects with other referring hospitals and can be seen as a starting point of regional integration with other referring hospitals.

As multiple interventions have been implemented in this collaboration project, it is difficult to identify strong correlations between individual interventions and both improved clinical outcomes and higher patient satisfaction. In general, redefining of the scheduling of physicians and the decision to increase physician staffing might have had positive effects on several endpoints [22]. In a recent report concerning the rate of readmissions [13], quality improvement efforts to improve inpatient care and the coordination of transitional care can prevent many unnecessary hospital readmissions. On the other hand, in a 2007 systematic review [23], only half of studies concluded that better hospital-level processes were associated with lower mortality; 18% found results in the opposite direction.

In addition to the collaboration project, there have been quality improvement projects in both SJG Weert and the Catharina cardiac centre that might have influenced the results of this study. For instance, in Catharina cardiac centre, improvement projects have been implemented within the cardiothoracic surgery department with positive effects on outcomes [24].

The primary means of assessing how patients feel about the care they receive in a health care setting is measurement of patient satisfaction. Patients have different views from health professionals when judging the quality of care and services [25]. We have used the results of a patient satisfaction survey to further improve care management and promote the quality of outcomes of referred patients. The present report shows a higher level of patient satisfaction in 2015 compared to the evaluation in 2013. In both hospitals, the degree of patient satisfaction about the personal contact between patient and physician was significantly improved. This may be a reflection of better and efficient planning, less work pressure, and consequently more attention for the individual patient.

### Strengths and limitations

One of the major strengths of this study was the focus on different kinds of quality indicators, namely clinical outcomes and patient satisfaction. A limited, but well-defined and widely accepted set of patient-relevant outcome measures has been included. A whole range of clinical outcomes is covered: survival, process of recovery (e.g. complications) and sustainability of health (Porter, 2010). As a result of an intensified collaboration in the care chain, improvement on both kinds of indicators have been observed.

The present study also has its limitations. First, multiple interventions have been implemented. Further research is required to identify correlations between individual interventions and improved clinical outcomes and higher patient satisfaction. Second, we have used a combined end-point to assess effects on clinical outcomes. This was done to increase power, but has the disadvantage of losing specific information regarding clinical outcomes. Third, the obtained results regarding patient satisfaction should be generalized with caution. The questionnaire was based on the standardized and validated Consumer Quality Index (CQI) [26, 27], but was not validated in the format it was used. Besides, only among a subsample of patients included in the outcome analyses the patient satisfaction questionnaire was conducted. This method, in combination with the low response rate, resulted in a lower number of patients included in the analyses regarding patient satisfaction. On the other hand, the included number of patients was sufficient to demonstrate statistically significant differences. Forth, further follow-up is needed to confirm our results and long-term outcome measures should be included. Finally, we did not include the role of the general practitioner in the improvement measures of the care chain, which is equally important and must be considered.

## Conclusions

In our study, a cardiac centre and a referring hospital seemed to succeed in improving patient-relevant clinical outcomes and patient satisfaction as a result of enhanced collaboration and first steps regarding regional integration. The results of our study indicate that there is reason to encourage other healthcare providers to intensify collaboration between cardiac centres and referring hospitals. One can assume that this would apply also to other – non-cardiac – interventions.

## Supplementary information

**Additional file 1.** Questionnaire patient satisfaction

## Abbreviations

CABG: Coronary artery bypass graft
CAD: Coronary artery disease
CVA: Cerebrovascular accident
CQI: Consumer quality index
DSWI: Deep sternal wound infection
FTE: Full-time equivalents
ICHOM: International consortium for health outcomes measurement
LVEF: Left ventricular ejection fraction
MI: Myocardial infarction
OR: Odds ratio
PCI: Percutaneous coronary intervention
SJG: St. Jans Gasthuis
VBHC: Value-based healthcare
