# Supplementary material for: Improving clinical outcomes and patient satisfaction among patients with coronary artery disease: an example of enhancing regional integration between a cardiac centre and a referring hospital
Source: BMC Health Serv Res. 2020 Jun 3;20:494. doi: 10.1186/s12913-020-05352-w (PMC7268761; doi:10.1186/s12913-020-05352-w)
Supplement: Supplementary file 1 — Additional file 1. Questionnaire patient satisfaction [file 12913_2020_5352_MOESM1_ESM.doc]

**
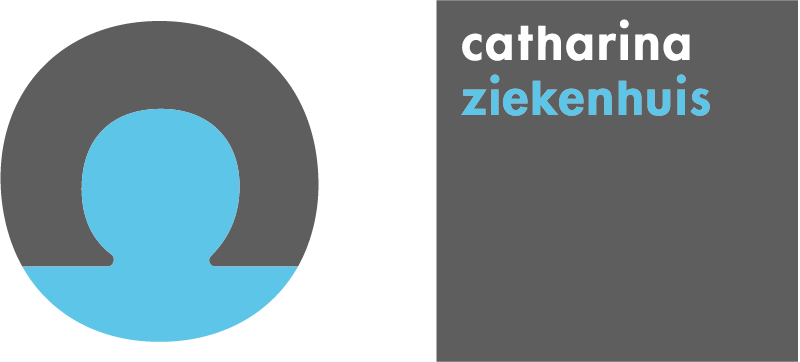

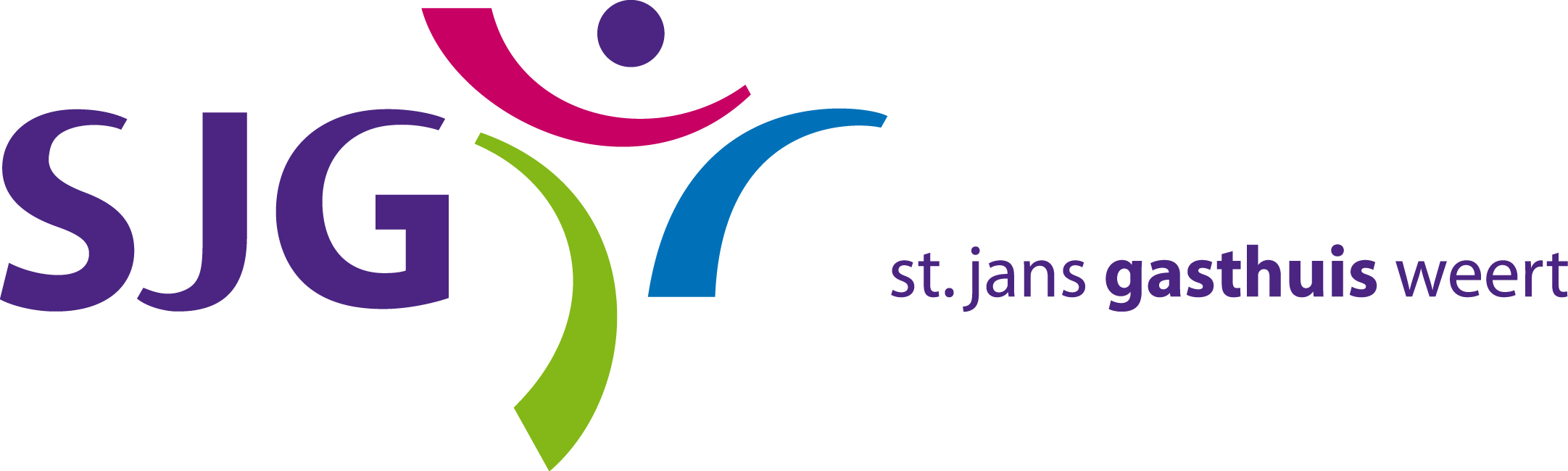
**

**Questionnaire patient satisfaction**

**General questions**

*In this questionnaire, we ask for your opinion, with 1 being the lowest and 10 being the highest grade. You can also select “not applicable” (n/a).*

Was your referral from St. Jans Gasthuis to Catharina Hospital a planned or an emergency transfer?

O Planned

O Emergency

|  | **1** | **2** | **3** | **4** | **5** | **6** | **7** | **8** | **9** | **10** | **n/a** |
| --- | --- | --- | --- | --- | --- | --- | --- | --- | --- | --- | --- |
| To what extent are you satisfied with the information you received / the communication between you and your hospital during your entire care process? | 🔾 | 🔾 | 🔾 | 🔾 | 🔾 | 🔾 | 🔾 | 🔾 | 🔾 | 🔾 | 🔾 |
| To what extent did the treatment correspond with what you had been told in advance? | 🔾 | 🔾 | 🔾 | 🔾 | 🔾 | 🔾 | 🔾 | 🔾 | 🔾 | 🔾 | 🔾 |

**Education and education material**

|  | **1** | **2** | **3** | **4** | **5** | **6** | **7** | **8** | **9** | **10** | **n/a** |
| --- | --- | --- | --- | --- | --- | --- | --- | --- | --- | --- | --- |
| To what extent are you satisfied with the verbal education prior to your treatment? | 🔾 | 🔾 | 🔾 | 🔾 | 🔾 | 🔾 | 🔾 | 🔾 | 🔾 | 🔾 | 🔾 |
| To what extent are you satisfied with the education material you received prior to your treatment? * ** N.B. You can skip this question if you have been referred urgently.* | 🔾 | 🔾 | 🔾 | 🔾 | 🔾 | 🔾 | 🔾 | 🔾 | 🔾 | 🔾 | 🔾 |
| To what extent are you satisfied with the verbal education after your treatment? | 🔾 | 🔾 | 🔾 | 🔾 | 🔾 | 🔾 | 🔾 | 🔾 | 🔾 | 🔾 | 🔾 |
| To what extent are you satisfied with the education material you received after your treatment? | 🔾 | 🔾 | 🔾 | 🔾 | 🔾 | 🔾 | 🔾 | 🔾 | 🔾 | 🔾 | 🔾 |

**Communication between St. Jans Gasthuis and Catharina Hospital**

|  | **1** | **2** | **3** | **4** | **5** | **6** | **7** | **8** | **9** | **10** | **n/a** |
| --- | --- | --- | --- | --- | --- | --- | --- | --- | --- | --- | --- |
| To what extent was it clear to you to whom you could go with your questions at what time? | 🔾 | 🔾 | 🔾 | 🔾 | 🔾 | 🔾 | 🔾 | 🔾 | 🔾 | 🔾 | 🔾 |
| To what extent did the two hospitals possess the same information? | 🔾 | 🔾 | 🔾 | 🔾 | 🔾 | 🔾 | 🔾 | 🔾 | 🔾 | 🔾 | 🔾 |

**Communication with the patient’s general practitioner (GP)**

|  | **1** | **2** | **3** | **4** | **5** | **6** | **7** | **8** | **9** | **10** | **n/a** |
| --- | --- | --- | --- | --- | --- | --- | --- | --- | --- | --- | --- |
| To what extent are you satisfied with the communication between the medical specialists at St. Jans Gasthuis and your GP? | 🔾 | 🔾 | 🔾 | 🔾 | 🔾 | 🔾 | 🔾 | 🔾 | 🔾 | 🔾 | 🔾 |
| To what extent are you satisfied with the communication between the medical specialists at Catharina Hospital and your GP? | 🔾 | 🔾 | 🔾 | 🔾 | 🔾 | 🔾 | 🔾 | 🔾 | 🔾 | 🔾 | 🔾 |

**Access time**

|  | **1** | **2** | **3** | **4** | **5** | **6** | **7** | **8** | **9** | **10** | **n/a** |
| --- | --- | --- | --- | --- | --- | --- | --- | --- | --- | --- | --- |
| To what extent are you satisfied with the time you had to wait until you could go to St. Jans Gasthuis with your complaints? | 🔾 | 🔾 | 🔾 | 🔾 | 🔾 | 🔾 | 🔾 | 🔾 | 🔾 | 🔾 | 🔾 |
| To what extent are you satisfied with the time between your referral by the physician at St. Jans Gasthuis and the moment you could go to the Catharina Hospital? | 🔾 | 🔾 | 🔾 | 🔾 | 🔾 | 🔾 | 🔾 | 🔾 | 🔾 | 🔾 | 🔾 |

**Quality of care**

|  | **1** | **2** | **3** | **4** | **5** | **6** | **7** | **8** | **9** | **10** | **n/a** |
| --- | --- | --- | --- | --- | --- | --- | --- | --- | --- | --- | --- |
| To what extent are you satisfied with the quality of care provided at the outpatient clinic at St. Jans Gasthuis? | 🔾 | 🔾 | 🔾 | 🔾 | 🔾 | 🔾 | 🔾 | 🔾 | 🔾 | 🔾 | 🔾 |
| To what extent are you satisfied with the quality of care provided at the outpatient clinic at Catharina Hospital? | 🔾 | 🔾 | 🔾 | 🔾 | 🔾 | 🔾 | 🔾 | 🔾 | 🔾 | 🔾 | 🔾 |
| To what extent are you satisfied with the quality of care provided at the nursing ward in St. Jans Gasthuis? | 🔾 | 🔾 | 🔾 | 🔾 | 🔾 | 🔾 | 🔾 | 🔾 | 🔾 | 🔾 | 🔾 |
| To what extent are you satisfied with the quality of care provided at the nursing ward in Catharina Hospital? | 🔾 | 🔾 | 🔾 | 🔾 | 🔾 | 🔾 | 🔾 | 🔾 | 🔾 | 🔾 | 🔾 |

**Unforeseen events or complications**

Did any unforeseen events or complications occur?

O yes

O no *(continue with question block “Hospital stay” on the next page)*

|  | **1** | **2** | **3** | **4** | **5** | **6** | **7** | **8** | **9** | **10** | **n/a** |
| --- | --- | --- | --- | --- | --- | --- | --- | --- | --- | --- | --- |
| To what extent are you satisfied with the treatment of these unforeseen events / complications in St. Jans Gasthuis? | 🔾 | 🔾 | 🔾 | 🔾 | 🔾 | 🔾 | 🔾 | 🔾 | 🔾 | 🔾 | 🔾 |
| To what extent are you satisfied with the treatment of these unforeseen events / complications in Catharina Hospital? | 🔾 | 🔾 | 🔾 | 🔾 | 🔾 | 🔾 | 🔾 | 🔾 | 🔾 | 🔾 | 🔾 |

**Hospital stay**

|  | **1** | **2** | **3** | **4** | **5** | **6** | **7** | **8** | **9** | **10** | **n/a** |
| --- | --- | --- | --- | --- | --- | --- | --- | --- | --- | --- | --- |
| To what extent do you appreciate the way in which you were welcomed at the outpatient clinic at St. Jans Gasthuis? | 🔾 | 🔾 | 🔾 | 🔾 | 🔾 | 🔾 | 🔾 | 🔾 | 🔾 | 🔾 | 🔾 |
| To what extent do you appreciate the way in which you were welcomed at the outpatient clinic at Catharina Hospital? | 🔾 | 🔾 | 🔾 | 🔾 | 🔾 | 🔾 | 🔾 | 🔾 | 🔾 | 🔾 | 🔾 |
| To what extent do you appreciate your stay at the nursing ward in St. Jans Gasthuis? | 🔾 | 🔾 | 🔾 | 🔾 | 🔾 | 🔾 | 🔾 | 🔾 | 🔾 | 🔾 | 🔾 |
| To what extent do you appreciate your stay at the nursing ward in Catharina Hospital? | 🔾 | 🔾 | 🔾 | 🔾 | 🔾 | 🔾 | 🔾 | 🔾 | 🔾 | 🔾 | 🔾 |

**Overall grade**

|  | **1** | **2** | **3** | **4** | **5** | **6** | **7** | **8** | **9** | **10** | **n/a** |
| --- | --- | --- | --- | --- | --- | --- | --- | --- | --- | --- | --- |
| Which grade do you award the overall care provided by St. Jans Gasthuis? | 🔾 | 🔾 | 🔾 | 🔾 | 🔾 | 🔾 | 🔾 | 🔾 | 🔾 | 🔾 | 🔾 |
| Which grade do you award the overall care provided by Catharina Hospital? | 🔾 | 🔾 | 🔾 | 🔾 | 🔾 | 🔾 | 🔾 | 🔾 | 🔾 | 🔾 | 🔾 |

**Personal contact**

|  | **1** | **2** | **3** | **4** | **5** | **6** | **7** | **8** | **9** | **10** | **n/a** |
| --- | --- | --- | --- | --- | --- | --- | --- | --- | --- | --- | --- |
| How do you rate the personal contact between you and your doctor at St. Jans Gasthuis? | 🔾 | 🔾 | 🔾 | 🔾 | 🔾 | 🔾 | 🔾 | 🔾 | 🔾 | 🔾 | 🔾 |
| How do you rate the personal contact between you and your doctor at Catharina Hospital? | 🔾 | 🔾 | 🔾 | 🔾 | 🔾 | 🔾 | 🔾 | 🔾 | 🔾 | 🔾 | 🔾 |

**Suggestions**

Do you have any suggestions on how St. Jans Gasthuis and Catharina Hospital can improve the care provided to you?

**Thank you very much for answering the questionnaire.**
